# A case report of hepatocarcinoma-originated pericardial malignancy

**DOI:** 10.3389/fcvm.2025.1643805

**Published:** 2025-09-18

**Authors:** Bin Wu, Zhixiao Wang, Weizhe Lin, Jian Xiong, Chaoyong He

**Affiliations:** Department of Cardiology, Taihe Hospital, Hubei University of Medicine, Shiyan, China

**Keywords:** hepatocellular carcinoma, cardiac metastases, diagnostic imaging—methods, lenvatinib, camrelizumab

## Abstract

Cardiac tumors constitute an exceptionally rare neoplastic entity posing significant diagnostic challenges. We report a 55-year-old female patient without prior oncologic history who presented with acute-onset bilateral lower extremity edema progressing over 72 h. Transthoracic echocardiography demonstrated a pericardial mass with concomitant hemorrhagic pericardial effusion. Subsequent magnetic resonance imaging and systemic positron emission tomography localized the lesion to the right bottom of the heart. Surgical exploration suggested a cardiac occupancy as an irregular, fish-flesh-like soft tissue mass, pathology biopsy was performed suggesting a malignant tumour of epithelial origin, and immunohistochemistry was suggestive of hepatic origin. The patient received combination therapy comprising programmed death-1 inhibitor camrelizumab (200 mg via intravenous infusion every 21 days) and oral lenvatinib (8 mg once daily). Serial contrast-enhanced computed tomography of the thorax and abdomen demonstrated progressive metastatic dissemination with malignant pleural and peritoneal effusion formation. Despite therapeutic intervention, the patient ultimately experienced disease progression culminating in mortality.

## Introduction

While malignant neoplasms theoretically possess metastatic potential to all organ systems, including the heart, cardiac metastases remain uncommon in clinical practice. The most frequent primary malignancies associated with cardiac metastasis include cutaneous melanoma, pulmonary carcinoma (1). Few cases of liver cancer as the source of metastasis have been reported (2, 3).

## Case description

**Figure 1:**
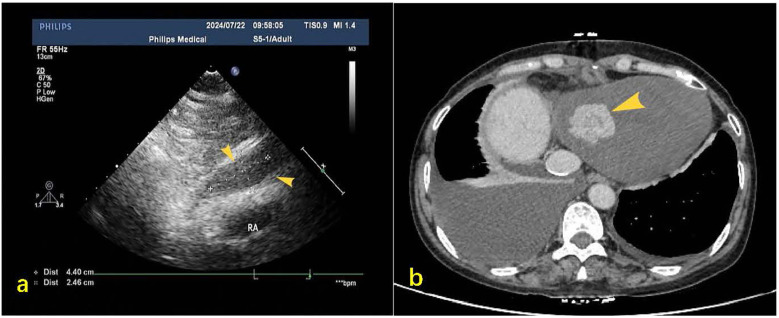
**(a)** Post-pericardiocentesis echocardiographic follow-up revealed a hypoechoic mass measuring approximately 44 × 25 mm in the pericardial cavity at the base of the right heart during subxiphoid examination (yellow arrow). **(b)** Contrast-enhanced CT on mediastinal window settings reveals an irregular soft tissue mass located at the base of the heart and the inferior aspect of the right atrium, measuring approximately 4.0 × 2.1 cm (indicated by the yellow arrow), exhibiting heterogeneous enhancement.

**Figure 2:**
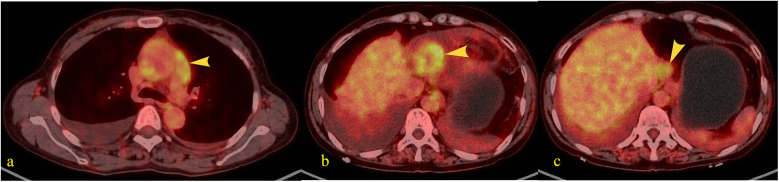
Patients fasted for over 6 h prior to intravenous injection of the radiotracer (¹⁸F-FDG). After resting quietly for 60 min, whole-body PET and CT tomographic imaging were performed. PET images underwent attenuation correction and iterative reconstruction. Both PET and CT images were displayed in multi-planar and multi-frame formats, demonstrating clear image quality. **(a)** A band-like soft tissue density lesion (yellow arrow) was observed adjacent to the ascending aorta (pericardial region), with a CT value of approximately 37 HU. This lesion demonstrated intense radiotracer uptake, exhibiting a maximum standardized uptake value (SUVmax) of 4.2–5.2. **(b)** A soft tissue density mass (yellow arrow) measuring approximately 4.6 × 3.4 cm was identified along the inferior right cardiac border (pericardial region), demonstrating intense radiotracer uptake with an SUVmax of 5.6. **(c)** A roundish nodule (yellow arrow) was identified at the superior margin of the hepatic caudate lobe, demonstrating heterogeneous density with a central hypodense area and measuring approximately 2.5 cm in diameter. Intense radiotracer uptake was observed within the lesion, with a maximum standardized uptake value (SUVmax) of 4.0.

**Figure 3:**
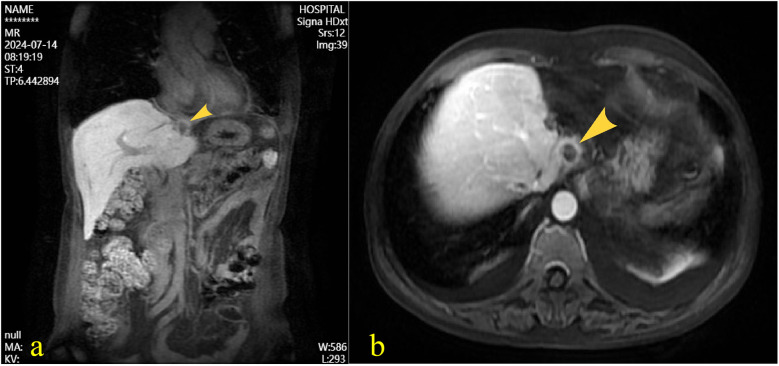
Following intravenous administration of gadoxetate disodium, magnetic resonance imaging of the liver revealed a well-defined roundish abnormal signal near the diaphragmatic surface of the left hepatic lobe. The lesion demonstrated mixed iso-to-hyperintensity on T1-weighted imaging, with signal drop-out on opposed-phase sequences, and iso-to-hyperintense signal on T2-weighted imaging. The longest diameter measured approximately 1.2 cm. **(a)** coronal view, **(b)** Axial view.

**Figure 4:**
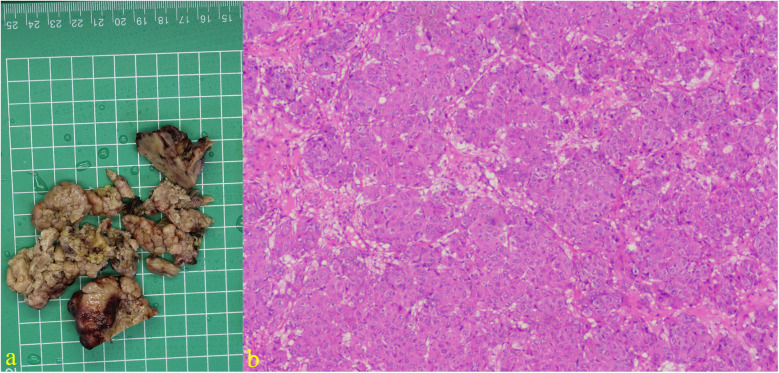
Excision specimen showing cardiac tumor: **(a)** Immunohistochemical results of tumor tissue suggest that AFP, CK (P), CK8/18, CEA, and caldesmon are positive, CK7, MDM2, SMA, desmin, MyoD1, myogenin, myoglobin, hepa, vimentin, SALL4, Glypican-3, calretinin are negative. **(b)** Hematoxylin and eosin staining (×200).

A 55-year-old female patient with a 2-week history of bilateral lower extremity edema was admitted to our department (July 4, 2024), who had a history of left liver lobectomy for intrahepatic bile duct stones. Laboratory tests on admission showed that the alpha-fetoprotein (AFP) value was 225 times higher than the upper limit of the normal reference range (0–15 ug/L), and the carbohydrate antigen (CA125) value was 9 times higher than the upper limit of the normal reference range (0–20 U/mL). Large amounts of pericardial effusion were seen on cardiac ultrasonography. Following the evacuation of the effusion, a faint echogenic mass of approximately 4.4 × 2.5 cm was observed in the pericardial cavity near the bottom of the right heart. (Figure 1a, July 22, 2024). And, an enhanced computed tomography (CT) revealed an intrapericardial mass measuring around 4.0 × 2.1 cm and a right pleural effusion, but no liver mass was detected (Figure 1b, July 6, 2024). Subsequently, the systemic positron emission tomography (PET) examination showed an abnormal uptake of 18-fluoro-2-deoxyglucose in the areas of the upper margin of the hepatic caudate lobe, the right margin of the heart and the banded soft tissue beside the ascending aorta (Figure 2, July 11, 2024). Utilizing hepatobiliary specific magnetic resonance contrast agents in enhanced magnetic resonance imaging (MRI), we discovered a circular enhancement in the left hepatic lobe close to the diaphragm surface with a diameter of 0.6 cm (Figure 3, July 14, 2024). Piercing fluids from both pericardial and thoracic cavities were bloody exudate, and no malignant cells were detected. On July 24th, 2024, surgical exploration was performed and an irregular and sessile fish-fleshed-like soft tissue mass in the pericardium near the bottom of the heart was found, measuring approximately 4 cm × 5 cm. Meanwhile, a tissue with a size of about 8 cm*7.2 cm*1.6 cm was removed during the operation, and a cardiac malignant tumor was identified by pathological biopsy. The immunohistochemistry results indicated a metastatic hepatic adenocarcinoma or metastasis of hepatocellular carcinoma (Figure 4). During surgical exploration the mass was found not to invade the myocardium but demonstrated ill-defined margins with the diaphragm. Subsequent diaphragmatic incision revealed an intact hepatic surface, suggesting low probability of direct hepatocellular carcinoma extension to the heart. Intraoperative identification of multiple soft tissue masses in the pericardial transverse and oblique sinuses prompted cytoreductive surgery for widespread metastases. Postoperative therapy with Lenvatinib combined with camrelizumab was initiated. During monthly outpatient follow-ups, serially elevated AFP levels reaching 7,920 times the upper reference limit were observed (Supplementary Data Figure S1). Surveillance CT demonstrated progressive massive pleural effusion with newly developed thoracic and abdominal cavity metastases. The patient received palliative care and died on January 3, 2025.

## Discussion

The open thoracic biopsy specimen demonstrated characteristic nest-like architecture consistent with histologic features of epithelial-derived neoplasia. Microscopic examination revealed moderate to severe cytologic atypia, including an elevated nuclear-to-cytoplasmic ratio and brisk mitotic activity (15 mitoses per 10 high-power fields at ×200 magnification) in hotspot areas, accompanied by geographic necrosis, meeting the diagnostic criteria for proliferatively active malignant tumors. Immunophenotypic analysis demonstrated positivity for AFP, pan-cytokeratin (CK-pan), and cytokeratin 8/18. Special staining revealed a bile canaliculus-like distribution pattern of carcinoembryonic antigen (CEA), collectively indicative of hepatocellular carcinoma (HCC). Although HepPar-1 and Glypican-3 were negative, the immunoprofile, when interpreted in the context of tumor differentiation, aligns more closely with poorly differentiated hepatocellular carcinoma. Proliferative activity assessment showed a Ki-67 labeling index of 10%. In conjunction with the mitotic count and extent of necrosis, these features indicate a tumor with significant aggressive potential. Among other negative indicators, the expression patterns of cytokeratin 7 and calretinin effectively excluded the two most important differential diagnoses—cholangiocarcinoma and mesothelioma. Sal-like protein 4 (SALL4) may help temporarily exclude germ cell tumors, while other myogenic markers can aid in ruling out various types of sarcomas. By integrating the biopsy specimen, immunohistochemical results, and imaging localization, we identified the primary focus in the liver.

Cardiac tumors presentation of cardiac masses varies depending on the size, location of the mass, blocking possibility and relationship to the heart's anatomy. Treatment of cardiac tumors differs based on the pathological type of the tumor and whether there are metastases. The common site of cardiac metastasis is the pericardium followed by the epicardium and myocardium, and the most common origins of metastatic tumors, in descending order, are pleural mesothelioma, melanoma, adenocarcinoma of the lungs, undifferentiated carcinoma, lung carcinoma, breast carcinoma, ovarian carcinoma, lymphoproliferative neoplasms, fine bronchial alveolar cancer, stomach cancer, kidney cancer and pancreatic cancer4. Primary benign/non-metastatic malignant cardiac tumors (excluding rhabdomyomas) require *en bloc* resection with negative margins for cure. Rhabdomyomas often regress spontaneously or respond to mTORC1 inhibitors, avoiding surgery. Metastatic cardiac tumors (primary with systemic spread or secondary metastases) necessitate systemic therapy, reserving surgery for palliation. Given the complexity of cardiac tumors, treatments are optimally formulated through multidisciplinary deliberations, and even in such cases, the prognosis is frequently dismal (4).

HCC ranks as the sixth most prevalent cancer worldwide and the fourth leading cause of cancer-related mortality. Cirrhosis from any etiology constitutes the strongest risk factor for HCC, including chronic alcohol abuse, diabetes, hepatitis associated with metabolic liver diseases, and viral hepatitis infections (5). HCC represents the predominant primary hepatic malignancy, accounting for 75%–85% of all liver cancers. Extrahepatic metastases predominantly involve the pulmonary system, intra-abdominal lymph nodes, and osseous structures, whereas cardiac involvement remains exceptionally rare, with reported incidence below 1% in autopsy studies (6, 7). Current therapeutic strategies are stratified according to the Barcelona Clinical Liver Cancer (BCLC) staging system, which integrates tumor characteristics, liver functional reserve, and performance status. For early-stage disease (BCLC 0/A), curative-intent modalities including anatomical hepatectomy, liver transplantation, and image-guided tumor ablation (radiofrequency or microwave) constitute the standard of care. Intermediate-stage HCC (BCLC B) is optimally managed with transarterial chemoembolization (TACE), a locoregional therapy combining embolization and cytotoxic drug delivery. Advanced-stage presentations (BCLC C/D) require systemic pharmacotherapy, with first-line options comprising multi-kinase inhibitors (sorafenib, lenvatinib) and immune checkpoint inhibitors (atezolizumab/bevacizumab combination) (8).

Thorough review of the patient's medical history revealed no established HCC risk factors, including chronic alcohol abuse, metabolic disorders (diabetes mellitus/dyslipidemia), or prior HCC diagnosis. Extended virologic workup excluded hepatitis B/C infection. This clinical profile distinctly differs from documented cardiac metastasis cases of HCC, which typically present with established HCC histories and distinct hepatic space-occupying lesions (2, 3). This case presents a complex diagnostic challenge dominated by recurrent serous effusions. The intracardiac mass was initially undetected by echocardiography and chest CT, and was only identified following elevated AFP levels that prompted contrast-enhanced CT. Comprehensive imaging showed no hepatic focal lesions. Final diagnosis required advanced imaging and histopathological biopsy. Importantly, without characteristic liver imaging findings, conventional diagnostic approaches are prone to error. This underscores the need to include hepatocellular carcinoma in the differential diagnosis of unexplained pericardial effusion, even in the absence of typical hepatic manifestations.

Hepatic carcinoma predominantly metastasizes via hematogenous spread, followed by lymphatic dissemination. In this case with pericardial involvement, the cardiac anatomical milieu characterized by high-velocity blood flow, dynamic myocardial contractions, and dedicated coronary circulation concurrently maintains a suboptimal microenvironment for tumor cell survival (marked by elevated oxygen tension and deficient pro-growth factors). These combined barriers render hematogenous cardiac metastasis exceptionally challenging. Consequently, lymphatic dissemination or direct extension emerges as more plausible metastatic pathways, despite the cardiac lymphatic system being comparatively underdeveloped (6). This synergistic interplay of anatomical and molecular defenses underlies the extreme rarity of cardiac metastases. Direct invasion was ruled out due to the absence of radiographic evidence such as diaphragmatic discontinuity, irregular thickening, or a contiguous mass linking the liver and pericardium. Surgical exploration further confirmed a smooth diaphragmatic surface with no signs of tumor infiltration. Hematogenous spread was considered the most probable route. Tumor cells likely traveled through the hepatic vein into the inferior vena cava and then to the right atrium. This pathway is corroborated by PET-CT findings showing intense radiotracer uptake near the ascending aorta and along the inferior pericardial border of the right heart, regions characterized by high hemodynamic force. These areas are susceptible to tumor implantation due to favorable flow conditions. Additionally, tumor seeding into the pericardial and pleural cavities may explain the observed malignant effusions. Although PET-CT detected metastatic lymph nodes in mediastinal stations 2R, 4R, 4l, 7, and 9, primary lymphatic spread was excluded owing to the lack of nodal involvement in typical primary sites such as the hepatoduodenal ligament or retroperitoneum. The mediastinal nodal metastases are more consistent with secondary dissemination via pericardial lymphatic drainage following initial hematogenous spread to the heart.

Based on the current clinical and imaging data, the patient was diagnosed with metastatic HCC to the pericardium. The disease was classified as BCLC stage C, and curative surgical intervention was no longer feasible. Management focused on palliative measures, including systemic therapy, localized radiotherapy, symptomatic control of the pericardial mass, and drainage of pericardial effusion. Following pericardiocentesis and partial resection of the pericardial lesion, the patient began treatment with lenvatinib in combination with camrelizumab. However, subsequent outpatient monitoring revealed a consistent monthly increase in AFP levels. The patient later developed progressive symptoms including dyspnea, abdominal distension, decreased appetite, and peripheral edema. These complications significantly diminished both the patient's and family's confidence in the treatment regimen. The patient succumbed to the disease six months after initial diagnosis. Pericardial metastasis from hepatocellular carcinoma is exceptionally rare. This case not only contributes to the limited data available on such presentations but also documents survival outcomes under standard advanced HCC management involving lenvatinib and camrelizumab. A comparative review with existing literature has been included, offering valuable real-world evidence regarding the clinical course and treatment response in this uncommon metastatic setting.

In summary, we report an uncommon case of pericardial metastasis originating from occult hepatocellular carcinoma, which was only detected at the disseminated disease stage. Although surgical intervention offered temporary palliation of symptoms, the disease progressed rapidly, with a timeline from diagnosis to mortality of approximately six months. This is consistent with the poor prognosis typically associated with cardiac metastases from advanced malignancies, as demonstrated in previous cohort studies (4).

## Data Availability

The raw data supporting the conclusions of this article will be made available by the authors, without undue reservation.
