# Supplementary figures and images for: A case report of hepatocarcinoma-originated pericardial malignancy
Source: Front Cardiovasc Med. 2025 Sep 18;12:1643805. doi: 10.3389/fcvm.2025.1643805 (PMC12488558; doi:10.3389/fcvm.2025.1643805)

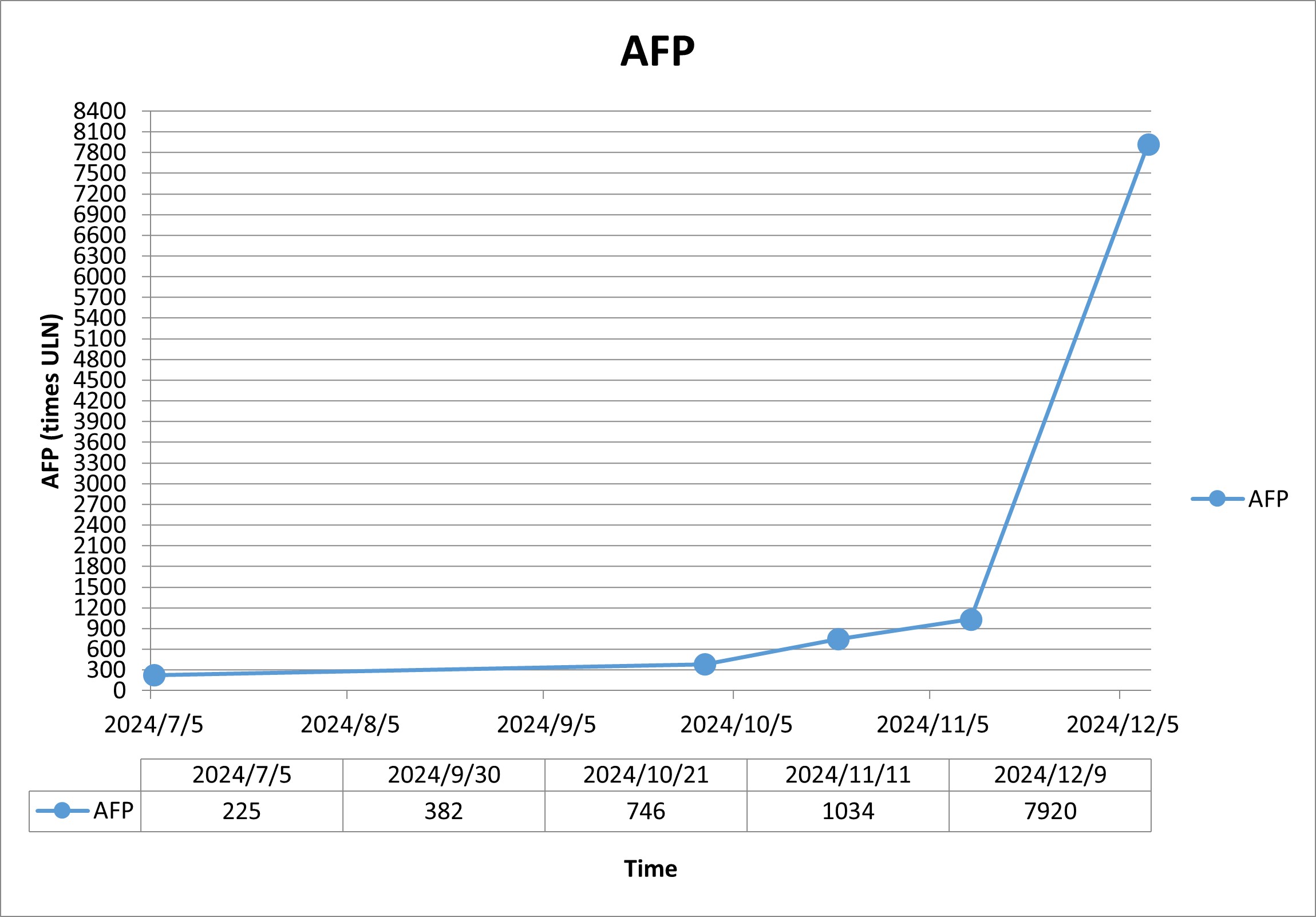

Supplement: Supplementary file 1 [file Image1.jpeg]
